# Serial electrocardiography to detect newly emerging or aggravating cardiac pathology: a deep-learning approach

**DOI:** 10.1186/s12938-019-0630-9

**Published:** 2019-02-12

**Authors:** Agnese Sbrollini, Marjolein C. De Jongh, C. Cato Ter Haar, Roderick W. Treskes, Sumche Man, Laura Burattini, Cees A. Swenne

**Affiliations:** 1 Cardiology Department, Leiden University Medical Center, PO Box 9600, 2300 RC Leiden, The Netherlands; 2 Information Engineering Department, Università Politecnica delle Marche, Via Brecce Bianche, 12, 60121 Ancona, Italy

**Keywords:** Serial electrocardiography, Vectorcardiography, Deep learning, Neural networks, Constructive algorithm

## Abstract

**Background:**

Serial electrocardiography aims to contribute to electrocardiogram (ECG) diagnosis by comparing the ECG under consideration with a previously made ECG in the same individual. Here, we present a novel algorithm to construct dedicated deep-learning neural networks (NNs) that are specialized in detecting newly emerging or aggravating existing cardiac pathology in serial ECGs.

**Methods:**

We developed a novel deep-learning method for serial ECG analysis and tested its performance in detection of heart failure in post-infarction patients, and in the detection of ischemia in patients who underwent elective percutaneous coronary intervention. Core of the method is the repeated structuring and learning procedure that, when fed with 13 serial ECG difference features (intra-individual differences in: QRS duration; QT interval; QRS maximum; T-wave maximum; QRS integral; T-wave integral; QRS complexity; T-wave complexity; ventricular gradient; QRS-T spatial angle; heart rate; J-point amplitude; and T-wave symmetry), dynamically creates a NN of at most three hidden layers. An optimization process reduces the possibility of obtaining an inefficient NN due to adverse initialization.

**Results:**

Application of our method to the two clinical ECG databases yielded 3-layer NN architectures, both showing high testing performances (areas under the receiver operating curves were 84% and 83%, respectively).

**Conclusions:**

Our method was successful in two different clinical serial ECG applications. Further studies will investigate if other problem-specific NNs can successfully be constructed, and even if it will be possible to construct a universal NN to detect any pathologic ECG change.

**Electronic supplementary material:**

The online version of this article (10.1186/s12938-019-0630-9) contains supplementary material, which is available to authorized users.

## Background

**Fig. 1:**
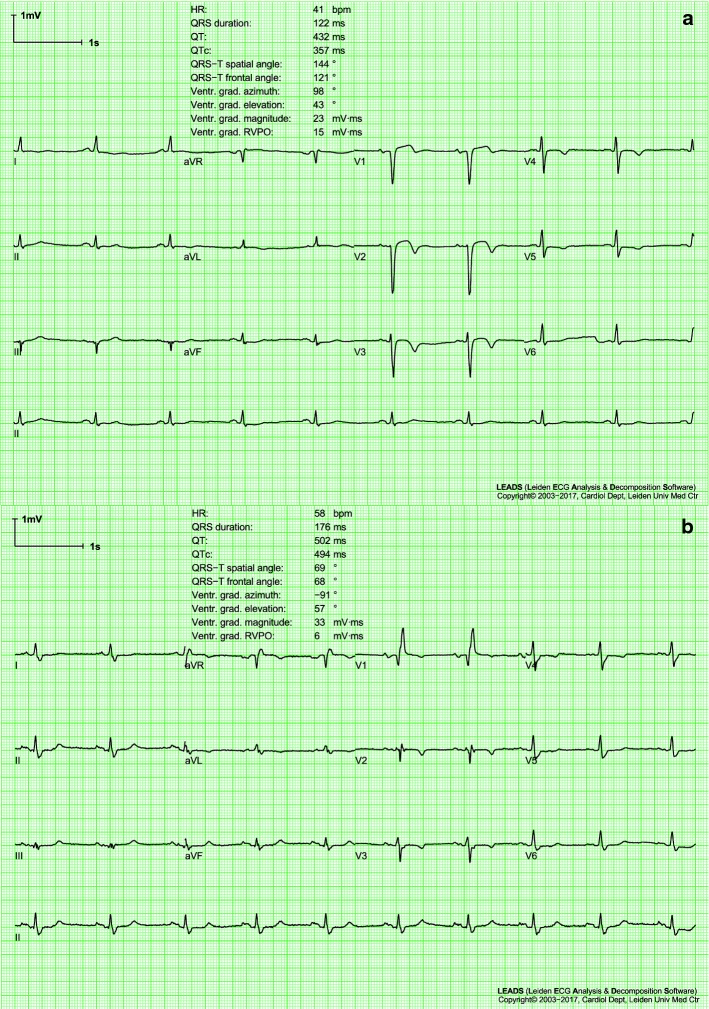
Two electrocardiograms (ECGs) of a case patient from the heart failure database (HFDB). The first ECG was made at baseline (**a**) and the second during follow-up (**b**). Both ECGs are standard 10-s 12-lead ECGs displayed according to the standard ECG display format. For each panel, the upper three traces show, multiplexed, 2.5 s of the four lead groups I/II/III, aVR/aVL/aVF, V1/V2/V3 and V4/V5/V6; instead, the longer trace displays continuously lead II, specifically used for rhythm analysis. A selection of measurements made by the LEADS program [13] is displayed in the upper part of each ECG page. See text for the clinical context and interpretation of these ECGs

The standard 10-s 12-lead electrocardiogram (ECG) is a diagnostic cornerstone of medicine. Serial electrocardiography is defined as the comparison of a newly made ECG with a previously made one, to look for possible changes. These changes are either used to detect new pathology or to verify the efficacy of a specific therapy or intervention. Serial ECG comparison is common clinical practice; usually, clinicians do this by visual assessment of the differences between two ECGs. Time distance between the two ECGs depends on their availability. Sometimes, serial ECGs are made in the setting of certain protocols (clinical research or check-up), other without any specific objective to perform a serial electrocardiographic analysis. An example of two serial ECGs is depicted in Fig. 1, that represents two standard 10-s 12-lead ECGs of a patient, made at baseline (panel a) and during follow-up (panel b). The two ECGs show impressive differences that clearly highlight the aggravation of the patient’s clinical condition (additional details on this case are provided in the "Results" section of this paper). Although visual comparison of two ECGs is normally performed by cardiologists in order to evaluate the aggravation of a cardiac pathology, studies reporting systematic application of approaches specifically developed for serial ECG analysis are still quite sporadic. To our knowledge, systematic serial ECG analysis has been previously applied to reveal pulmonary valve dysfunction in Fallot patients [1, 2] and to support the diagnosis of patients with suspected acute coronary syndrome [3].

As described before, serial electrocardiography aims at demonstrating a change in the clinical cardiac status of the patient. However, besides a clinical change, intra-subject ECG differences may also have a physiological or technical origin. Indeed, the ECG of a person changes with blood pressure, mental stress, body position, respiration rate, age and weight; additionally, irreproducible electrode positioning, specifically of the six precordial electrodes, is a major source of ECG variability. Together, ECG changes due to both physiological and technical causes constitute the “noise” of serial electrocardiography [4], whereas clinically relevant ECG changes represent the “data of interest”, the detection and the interpretation of which are limited by the signal-to-noise ratio, no matter whether serial ECG analysis is done by visual inspection or by computer analysis.

Some current commercial programs for automated computerized ECG analysis support serial electrocardiography interpretation. For example, the Glasgow program [5] compares an ECG with the previous ECG of the same patient when present in its database and produces a statement whether relevant changes occurred. Performance of this and other algorithms for serial ECG analysis have never been scrutinized. Automated serial ECG analysis has not reached the level of sophistication and validated performance that the algorithms for automated analysis of single ECG have achieved. Additionally, current algorithms for serial ECG analysis are rule-based and rigid. Typically based on threshold definitions, they consider only changes over threshold of a single feature, without considering single feature variations in time or the relative variations of several features for the identification of emerging or aggravating cardiac pathology. Because at present little can be said about which ECG changes are relevant in a specific clinical setting, a more flexible algorithm with learning abilities is needed.

Recently, several studies have demonstrated the potentiality of using machine learning for the prediction of cardiac pathology [6–10]. Aim of the present work is to present a novel approach that merges deep-learning classification methodology with serial electrocardiography. One important issue nowadays investigated in deep-learning is the design of algorithms for automated neural networks (NNs) construction [11, 12]. Our approach generates problem-specific NNs to diagnose newly emerging or aggravating cardiac pathology. We validated this approach by establishing its performance in the detection of newly emerging heart failure in post-infarction patients and acute ischemia in patients with a sudden short-lasting complete coronary occlusion. In order to confirm the superiority of flexible over rigid algorithms with learning ability, we analyzed the same populations with standard logistic regression, and compared the results obtained with our specifically-developed NN against those obtained by application of the logistic regression.

## Methods

### Method to construct a deep-learning neural network for serial electrocardiography

#### Feature selection

We compared two digital standard 10-s 12-lead resting ECGs of each patient: an initial baseline ECG (BLECG) and a follow-up ECG (FUECG). Each 12-lead ECG was converted into a vectorcardiogram (VCG), a coherently averaged beat was computed, after which 13 VCG features were computed that together represent the major cardiac electrical properties: QRS duration, QT interval, QRS maximum amplitude, T-wave maximum amplitude, QRS-integral vector magnitude, T-wave integral vector magnitude, QRS complexity, T-wave complexity, ventricular gradient vector, QRS-T spatial angle, heart rate, J-point vector and T-wave symmetry (computed as the ratio of the area between T-wave apex and end to the area between the J point and T-wave end) [13–15].

The VCG features are based on electrophysiological considerations: QRS duration is linked to intraventricular conduction; the QT interval is linked to intraventricular conduction and action potential duration; the maximum QRS amplitude is linked to ventricular mass; the maximum T-wave amplitude is sensitive to, e.g. ischemia and electrolyte abnormalities; the QRS and T-wave integrals are indexes of depolarization and repolarization dispersion, respectively; the QRS- and T-wave complexity measure the depolarization and repolarization processes complexity, respectively; the ventricular gradient measures heterogeneity of the action potential morphology distribution; the QRS-T spatial angle characterizes ECG concordance/discordance; heart rate partly expresses autonomic nervous system activity; and the J-point amplitude and T-wave symmetry also alter with ventricular ischemia. Together these VCG features cover that much aspects of electrical heart function that is difficult to imagine that electrical heart function could change without manifesting itself in a change in one or more of the 13 VCG features. Consequently, by subtracting the 13 BLECG VCG features from the corresponding 13 FUECG VCG features, the 13 difference features listed in Table 1 were obtained.

**Table 1:** List of the 13 difference features

#Feature	Abbreviation	Units	Description
1	$\Delta QRSdur$	ms	QRS-duration difference
2	$\Delta QT$	ms	QT-interval difference
3	$\Delta |{\overline{QRSmax}}|$	μV	Difference in maximal QRS-vector magnitude
4	$\Delta |{\overline{Tmax}}|$	μV	Difference in maximal T-vector magnitude
5	$\Delta |{\overline{QRSintg}}|$	mV·ms	QRS-integral vector magnitude difference
6	$\Delta |{\overline{Tintg}}|$	mV·ms	T-integral vector magnitude difference
7	$\Delta QRScmplx$	%	QRS-complexity difference
8	$\Delta Tcmplx$	%	T-wave complexity difference
9	$|\overline{\Delta VG}|$	mV·ms	Magnitude of the ventricular-gradient difference vector
10	$|\Delta SA|$	°	Magnitude of the QRS-T spatial-angle difference
11	$\Delta HR$	bpm	Heart-rate difference
12	$|\overline{\Delta J}|$	μV	Magnitude of J-vector difference vector
13	$\Delta Tsym$	%	T-wave symmetry difference

These 13 features are obtained from the subtraction of the 13 VCG features computed using the BLECG from the corresponding 13 VCG features computed using the FUECG

The difference features were chosen in such a way that, in variables where pseudo-normalization can occur (ventricular gradient, QRS-T spatial angle, J vector), the absolute value of the difference is considered [16]. All 13 difference features as defined above serve as input of our novel deep-learning classification method described below.

#### Repeated structuring and learning procedure for neural-network construction

To discriminate patients with altered clinical status from stable patients by serial ECG analysis, we developed a new method that automatically constructs NNs with a problem-specific architecture. For the purpose of learning and testing, we used ECG databases of patients with known clinically stable status, denominated controls, plus patients with a known pathological development during follow-up, denominated cases. Details about the ECG databases are described later in the "Methods" section. Databases were equally randomly divided into learning and testing datasets, containing data of both controls and cases. The learning datasets were further divided into a training dataset (in this study, 80% of the learning dataset) and a validation dataset (in this study, 20% of the learning dataset).

Our deep-learning classification algorithm consists of a supervised NN with 13 inputs (one for each difference feature) and 1 output. Output values range from 0 to 1, with 0 representing a control classification and 1 a case classification. Intermediate values indicate an uncertain classification, to be further processed using a case/control decision threshold. The NN consists of neurons with weights and biases between − 1 and + 1 and sigmoid activation functions. Its architecture is dynamically formed using the new repeated structuring and learning procedure (RS&LP), that we developed in order to handle this specific type of classification problems and that we describe here for the first time. The algorithm starts from an initial configuration of one hidden layer with 1 neuron (the minimal number of neurons per layer), which is initialized with random weights and bias. The maximal number of hidden layers is set at 3, while no maximal number of neurons per layer is set. The NN architecture is notated as horizontal vector in which the number of elements represents the number of layers, and the numerical value in each element represents the number of neurons in the corresponding layer.

Conventionally, for a given NN architecture, the learning algorithm adjusts neuron weights and biases according to the scaled-conjugate-gradients algorithm [17], to optimize the training set classification by minimizing a training-error function, computed as the normalized sum of the squared differences between estimated outputs and true classification values. Similarly, a validation-error function is computed for the validation dataset; it is expected to decrease monotonously during learning. In our learning algorithm, both the training-error and validation-error functions contain weights to compensate for the disproportion between the number of cases and controls [18]; in our algorithm, we assigned the inverse of the prevalence of the cases and controls in the dataset as their weights. The learning phase ends when the validation-error function starts to increase [19].

**Fig. 2:**
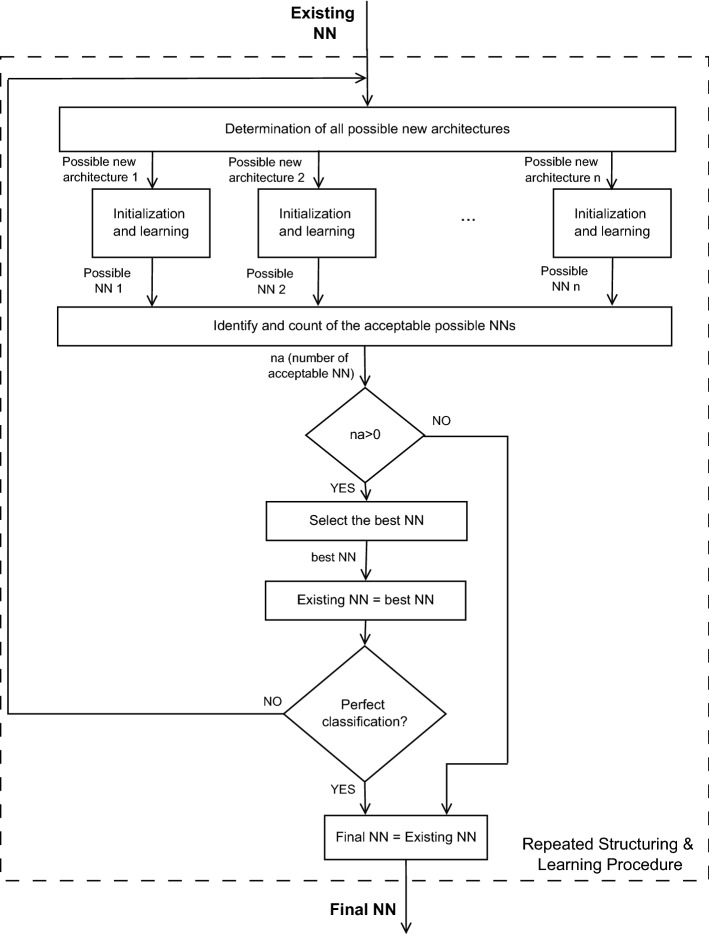
Flowchart of the repeated structuring and learning procedure (RS&LP) to construct a neural network (NN) for serial ECGs analysis

This conventional learning algorithm is integrated in our RS&LP, a supervised procedure that we designed to build a NN by alternating phases of structuring with phases of learning (Fig. 2). The RS&LP assumes that each new architecture contains the previous architecture plus one new neuron, and recursively applies the following 3 steps:

- Step1: determination of all possible new architectures;
- Step2: initialization of new neurons and learning of possible new architectures;
- Step3: selection of the new NN.

After Step3 is concluded, the procedure starts again from Step1; it ends only when a stopping criterion (see below) is met.

**Fig. 3:**
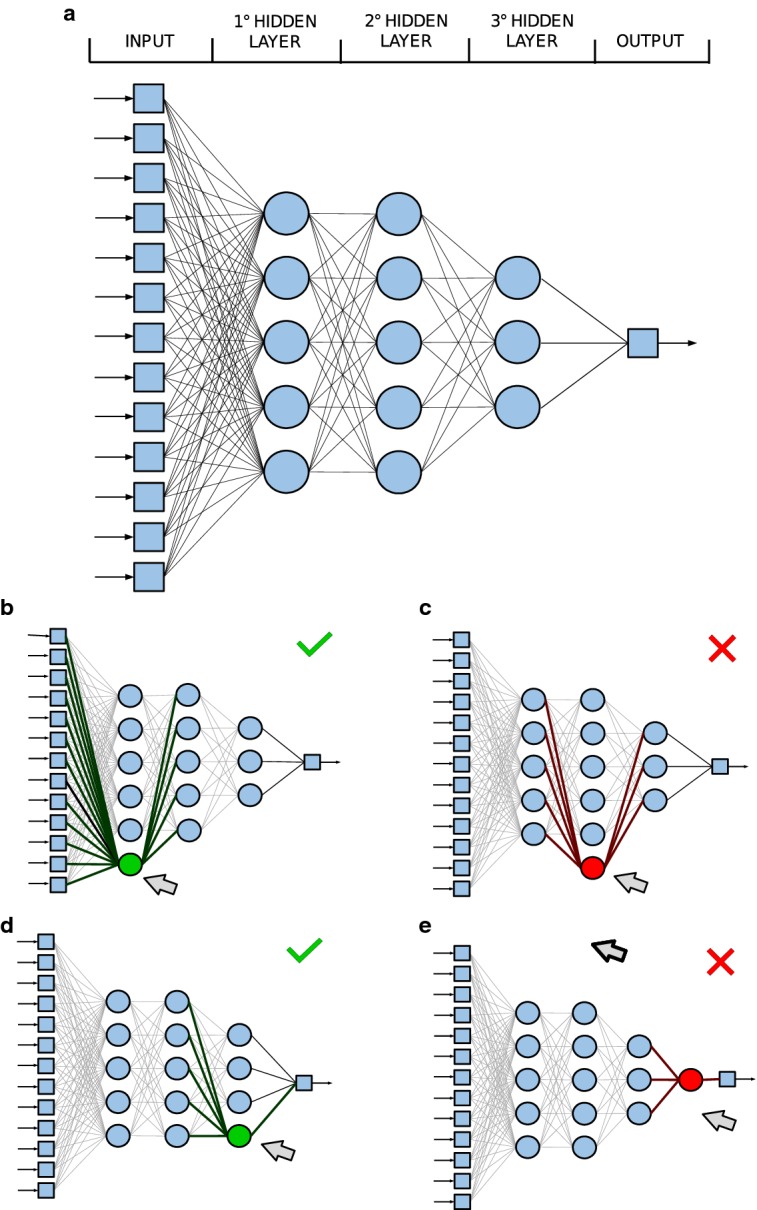
Example of determination of the possible new neural network (NN) architectures that can grow from a given NN (**a**) that emerged in the course of the repeated structuring and learning procedure (RS&LP). The new architecture will consist of the currently existing NN plus one additional neuron. The first attempt to create a new architecture consists of adding the extra neuron to the first hidden layer, this architecture is possible (**b**). The second attempt consists of adding an extra neuron to the second hidden layer, this architecture is not permitted because it would give the second hidden layer more neurons than the first hidden layer (**c**). The third attempt consists of adding the extra neuron to the third hidden layer, this architecture is possible (**d**). The fourth attempt consists of creating a new hidden layer with the extra neuron, this architecture is not permitted because the number of layers is limited to three (**e**). Hence, out of four attempts, two are successful (**b**,** d**) and will be evaluated in the next learning step

*Step1: Determination of the possible new architectures.* In each structuring cycle (see Fig. 3), possible new architectures are strategically built by adding one neuron to the existing NN. This can be done either by adding the neuron to an existing hidden layer or by creating an additional hidden layer consisting of the new neuron with the following constraints:

- The maximal number of hidden layers is three;
- The number of neurons in a given hidden layer may not be larger than the number of neurons in the previous hidden layer.

*Step2: Initialization of new neurons and learning of possible new architectures.* All possible new architectures keep the weights and biases of the neurons of the existing NN; only the new neuron is initialized with random weights and bias. A possible new architecture is acceptable only if new neurons increase training performance (decrease training error) after one iteration. If not, it undergoes a new neuron initialization or is rejected after 500 initializations. All accepted possible new architectures undergo the conventional learning process, at the end of which their validation error is either larger than the validation error of the existing NN (failure) or smaller/equal (success). In case of failure, the possible new NN is either re-initialized (at most 10 times) or rejected. Might all possible new architectures be rejected, the existing NN is kept as the final one and the RS&LP is stopped (first stopping criterion).

*Step3: selection of the new NN.* In case of success of one or more of the possible new NNs generated in step 2, the one with the lowest validation error is upgraded and becomes the new existing NN. Once a new existing NN has been selected, the RS&LP starts anew or stops if no misclassifications occurred in either the training or the validation dataset (second stopping criterion). This stopping criterion was incorporated to prevent the loss of generalization through overfitting [19].

#### Neural-network optimization

If the RS&LP is run two times on the same learning dataset, the resulting NNs will be different due to the random neuron initialization. In our implementation, 100 alternative NNs are constructed. For each of the 100 alternative NNs, the receiver operating characteristic (ROC) is obtained by varying the case/control decision threshold on the learning dataset, and the area under the curve (AUC) is computed. Finally, the NN with the largest learning AUC is selected.

### Clinical testing of neural network

We tested our RS&LP by constructing NNs for two different ECGs databases, a heart-failure database (HFDB) and an ischemia database (IDB).

The HFDB [16, 20] is composed of ECGs of patients who had experienced a myocardial infarction. An ECG, routinely made at least 6 months after the infarction and when the patients were clinically stable without any sign of heart failure, was selected as BLECG. Patients who remained stable were selected as controls, and a routinely made ECG recorded about 1 year after the BLECG was selected as FUECG. Patients who developed chronic heart failure were selected as cases; the ECG that was made when they presented themselves at the hospital for the first time with this newly arisen pathology was selected as FUECG. Overall, the HFDB contains 128 ECG pairs (47 cases and 81 controls). All ECGs were retrospectively selected from the digital ECG database of the Leiden University Medical Center. The HFDB was randomly equally split into a learning dataset (64 ECG pairs; 24 cases and 40 controls) and a testing dataset (65 ECG pairs; 24 cases and 41 controls). The learning dataset further split into a training dataset (54 ECG pairs; 20 cases and 34 controls) and a validation dataset (10 ECG pairs; 4 cases and 6 controls).

The IDB is composed of ECGs retrospectively selected from the digital ECG database of the Leiden University Medical Center (controls) and from the STAFF III ECG database [20–23] (cases). Control patients were outpatients of the cardiology department, selected on the availability of two digital ECG recordings made about one year apart (BLECG and FUECG, respectively). Cases had stable angina and underwent elective coronary angioplasty. In the STAFF III Study, balloon inflations, intended to widen the lumen of the stenotic vessel, were intentionally long, thus causing acute ischemia in the tissue distal from the occlusion. The BLECG and FUECG were taken immediately before and after 3 min of balloon occlusion, respectively. Overall, the IDB contains 482 ECG pairs (84 cases and 398 controls). For the purpose of our study, it was randomly equally split into a learning dataset (241 ECG pairs; 42 cases and 199 controls) and a testing dataset (241 ECG pairs; 42 cases and 199 controls). The learning dataset was further split into a training dataset (202 ECG pairs; 35 cases and 167 controls) and a validation dataset (39 ECG pairs; 7 cases and 32 controls).

All ECGs of both databases were analyzed by the Leiden ECG Analysis and Decomposition Software [13], that converts a 12-lead ECG into a VCG, computes the coherently averaged beat and determines QRS onset and offset (J point) and T-wave offset. Two independent ECG analysts reviewed the automatically-detected ECG landmarks and edited these when necessary. Using these landmarks, the 13 difference features were computed.

The present retrospective study on both HFDB and IDB is undertaken in compliance with the ethical principles of Helsinki Declaration and approved by the Leiden University Medical Center Medical Ethics Committee.

### Comparison of neural network with other methods

The NNs computed with the RS&LP ($\text {NN}_{RS \& LP}$) are computed after a many learning steps, alternating with structuring steps. Usually, the standard method to train a NN ($\text {NN}_{SM}$) with a fixed structure is to apply only one single training phase, according with the learning algorithm. In order to compare the RS&LP with the fixed-structure NN learning method, we trained $\text {NN}_{SM}$ that had the same architecture as the final $\text {NN}_{RS \& LP}$ in the conventional way, initializing the parameters of the $\text {NN}_{SM}$ and applying the learning phase only one single time while using the same data division and learning algorithm (scaled-conjugate-gradients algorithm [17]).

In the absence of data from literature, in order to confirm superiority of flexible over rigid algorithms with learning ability in serial ECG analysis, we compared the performance of the final $\text {NN}_{RS \& LP}$ with that of a standard logistic regression (LR) [18, 19, 24–26]. LR for case/control classification was constructed using the HFDB and IDB learning datasets. Cases and controls were weighted inversely to their prevalence [18]. When fed with the 13 difference features, LR computes a discriminating function (an exponential combination of the difference features) the value of which represents the classification value ranging from 0 (representing a control patient) to 1 (representing a case patient). As for the construction of the NNs, the discriminating function of LR was computed with the learning dataset.

### Statistics

The ECG and ROC feature distributions were described in terms of 50th [25th;75th] percentiles and compared using the Wilcoxon ranksum and DeLong’s tests [27]. $\text {NN}_{RS \& LP}$, $\text {NN}_{SM}$ and LR performances were quantified from the ROC curves of the learning and testing datasets in terms of AUC, 95$\%$ confidence intervals (CI) and the diagnostic accuracies (ACC; computed at the point of equal sensitivity and specificity), computing the ROC curves of the testing datasets. Statistical significance was set at 0.05.

### Implementation

**Fig. 4:**
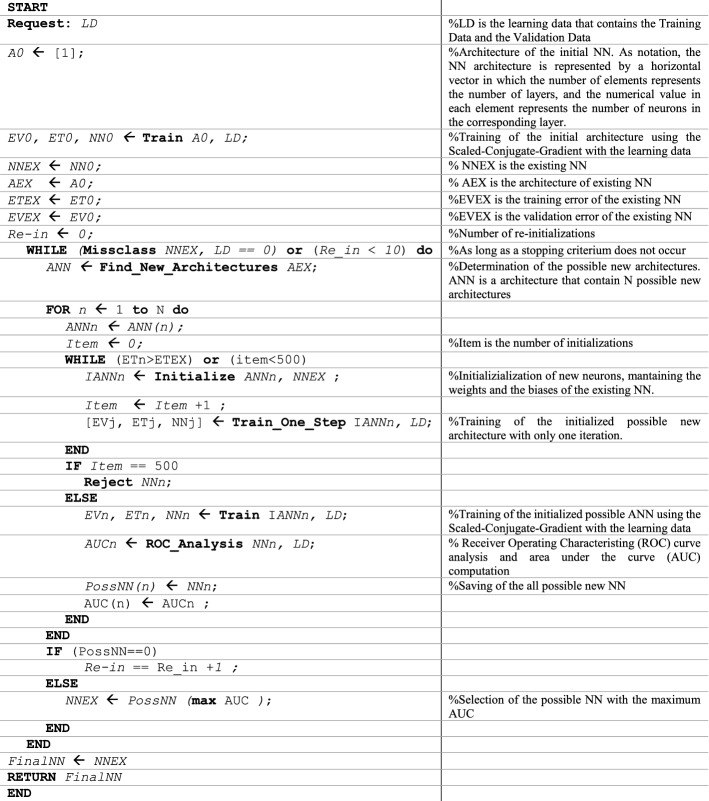
Pseudocode implementing the repeated structuring and learning procedure (RS&LP)

Programming was done in Matlab R2017a (The MathWorks, Natick, MA, USA). The flow-chart of the RS&LP has been represented in Fig. 2, showing the conceptual sequence of decisions needed to reach the final NN. Moreover, in order to better describe all steps of the procedure, Fig. 4 depicts the pseudocode of its implementation (Fig. 4, left column) with associated explanatory comments (Fig. 4, right column).

## Results

An example of two serial ECGs of a case patient from the HFDB is given in Fig. 1. The BLECG (panel a) of this patient was made six months after acute myocardial infarction. It has various pathological aspects, among which a long QRS duration (122 ms) and a negative T wave in various leads. Also the QRS-T spatial angle, which is the planar angle between the QRS- and T-wave axes, is pathological (144°) [28]. The FUECG (panel b) was made when the patient presented at the hospital for the first time with signs of heart failure. Also, this ECG is pathological and impressive differences with the BLECG can be seen; for example, the QRS width increased to 176 ms.

The quantitative characterization of the difference features distributions of both HFDB and IDB is reported in Table 2. The number of difference features that were statistically different between cases and controls was 9 in the HFDB ($\Delta$QRSdur, $\Delta |{\overline{Tmax}}|$, $\Delta |{\overline{QRSintg}}|$, $\Delta QRScmplx$, $\Delta Tcmplx$, $|\overline{\Delta VG}|$, $|\Delta SA|$, $\Delta HR$ and $|\overline{\Delta J}|$), and 8 in the IDB ($\Delta$QRSdur, $\Delta |{\overline{QRSmax}}|$, $\Delta |{\overline{QRSintg}}|$, $\Delta |{\overline{Tintg}}|$, $\Delta QRScmplx$, $|\Delta SA|$, $\Delta HR$ and $|\overline{\Delta J}|$).

**Table 2:** Quantitative characterization of the 13 difference features distributions in the HFDB and the IDB

	HFDB			IDB		
	Total	Controls	Cases	Total	Controls	Cases
	(N = 129)	(N = 81)	(N = 48)	(N = 482)	(N = 398)	(N = 84)
$\Delta QRSdur$ (ms)	0.0 [− 4.0;8.0]	0.0 [− 4.0;4.5]	4.0* [− 4.0;17.0]	0.0 [− 2.0;16.0]	0.0 [− 4.0;4.0]	8.0** [0.0;17.0]
$\Delta QT$ (ms)	1.0 [− 13.3;20.3]	− 1.0 [− 13.0;− 13.8]	7.5 [− 15.5;34.0]	2.0 [− 14.0;16.0]	2.5 [− 14.0;15.0]	0.0 [− 17.0;19.0]
$\Delta |{\overline{QRSmax}}|$ (μV)	− 26.0 [− 149.0;86.6]	− 34.2 [− 144.9;57.3]	− 11.9 [− 196.4;175.7]	− 21.1 [− 131.3;79.8]	− 12.1 [− 127.9;96.1]	− 38.9* [− 144.0;16.0]
$\Delta |{\overline{Tmax}}|$ (μV)	− 15.5 [− 65.7;39.7]	− 4.6 [− 42.0;40.0]	− 48.2* [− 133.9;33.2]	− 9.5 [− 68.1;53.5]	− 11.4 [− 68.0;45.4]	3.0 [− 70.4;87.8]
$\Delta |{\overline{QRSintg}}|$ (mV·ms)	0.4 [− 3.9;5.3]	− 0.76 [− 3.9;3.0]	3.5* [− 2.7;10.8]	− 0.0 [− 2.9;3.4]	− 0.3 [− 3.3;2.8]	1.7*** [− 1.5;9.1]
$\Delta |{\overline{Tintg}}|$ (mV·ms)	− 2.7 [− 13.3;7.1]	0.0 [− 10.6;7.1]	− 7.4 [− 19.2;8.1]	− 0.6 [− 10.6;8.9]	− 0.9 [− 10.7;7.5]	6.6** [− 10.4;30.1]
$\Delta QRScmplx$ (%)	0.3 [− 1.2;1.6]	− 0.0 [− 1.2;1.0]	1.3** [− 0.7;3.4]	0.1 [− 0.8;1.1]	0.0 [− 1.0;0.9]	0.6*** [− 0.3;2.8]
$\Delta Tcmplx$ (%)	0.4 [− 1.0;1.5]	0.0 [− 1.2;1.0]	1.6*** [0.2;4.1]	0.1 [− 1.2;1.4]	0.0 [− 1.1;1.1]	0.4 [− 3.6;4.4]
$|\overline{\Delta VG}|$ (mV·ms)	27.5 [16.1;41.8]	25.0 [14.6;36.2]	32.7* [18.1;50.0]	50.0 [37.5;65.2]	49.6 [38.0;63.0]	56.0 [33.4;86.6]
$|\Delta SA|$ (°)	13.3 [5.3;27.0]	9.1 [3.6;14.8]	31.7*** [14.9;56.0]	9.3 [4.0;17.2]	8.3 [3.7;15.1]	15.1*** [7.1;45.5]
$\Delta HR$ (bpm)	0.4 [− 5.2;6.1]	− 0.7 [− 5.3;4.6]	2.9* [− 2.2;10.0]	1.4 [− 4.3;6.5]	0.5 [− 4.9;5.8]	4.9*** [− 0.2;10.1]
$|\overline{\Delta J}|$ (μV)	24.6 [14.9;40.3]	21.5 [13.1;35.3]	32.7** [18.8;47.4]	25.7 [17.1;43.5]	22.8 [15.1;35.2]	68.6*** [41.5;122.7]
$\Delta Tsym$ (%)	− 0.1 [− 3.1;2.5]	− 0.2 [− 2.8;1.5]	0.0 [− 5.1;4.1]	0.2 [− 2.8;2.5]	0.2 [− 2.4;2.1]	0.8 [− 4.3;5.4]

Values are reported as percentiles 50th [25th;75th] percentiles

*,**,***: reflects P-value $<0.05$, $<0.01$, $<10^{-3}$, respectively, when comparing corresponding features computed for cases vs controls

**Fig. 5:**
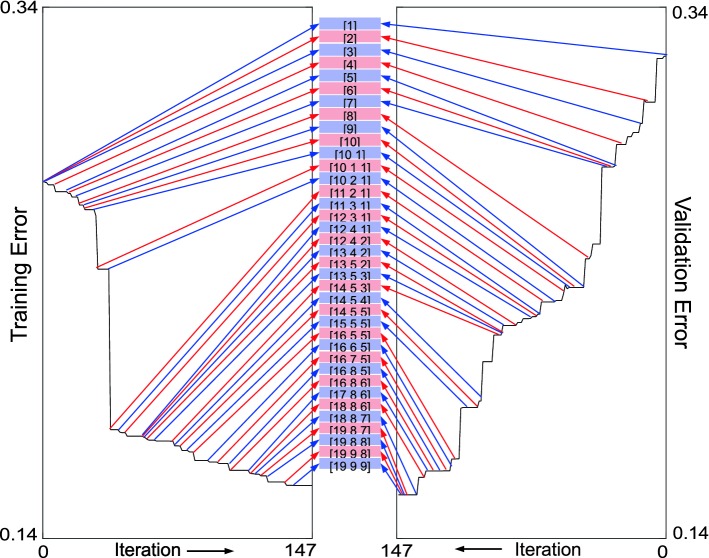
Example of the dynamic construction of a neural network (NN) by the repeated structuring and learning procedure (RS&LP) using the ischemia database (IDB). A total of 147 learning iterations of the scaled-conjugate-gradients algorithm, during which 37 new structures are created, leads from the initial architecture [1] to the final architecture [19 9 9]. The training error decreases monotonously (left panel). Some new architectures (e.g., [12 4 2]) are almost not contributing to a reduction of the training error, while others (e.g., [10 2 1]) strongly decrease the training error. With the introduction of a new architecture, the validation error (right panel) may increase in the first iteration (visible in the figure when the new structures [2] and [10 1] are initialized), but it has to decrease monotonously in following iterations. RS&LP stopped when the validation classification reached 100% correctness, yielding the structure [19 9 9]

As an example, Fig. 5 shows the dynamic construction of one alternative NN (not the final one) for the IDB by the RS&LP, from the initial architecture ([1]) to the final one ([19 9 9]).

The $\text {NN}_{RS \& LP}$ characteristics for the two databases obtained by our deep-learning method are reported in Table 3. Both $\text {NN}_{RS \& LP}$ efficiently discriminated patients with altered clinical status ($AUC\ge {83\%}$; $ACC\ge {75\%}$). The number of layers in the $\text {NN}_{RS \& LP}$ architectures was 3; the total number of neurons for the HFDB was 41, larger than the total number of neurons for the IDB, which was 21. Additionally, regarding the HFDB and the IDB the AUCs (84% and 83%, respectively) and the ACCs (75% and 76%, respectively) were comparable.

**Table 3:** $\text {NN}_{RS \& LP}$, $\text {NN}_{SM}$ and LRs characteristics for the HFDB and the IDB

	Architecture		HFDB	IDB
			[16 13 12]	[11 9 1]
$\text {NN}_{RS \& LP}$	Learning	AUC (%)	99	98
	Testing	AUC (%)	84	83
		CI (%)	[73–95]	[75–91]
		ACC (%)	75	76
$\text {NN}_{SM}$	Learning	AUC (%)	86	77
	Testing	AUC (%)	83	73
		CI (%)	[72–94]	[60–87]
		ACC (%)	75	67
LR	Learning	AUC (%)	89	88
	Testing	AUC (%)	61	77
		CI (%)	[46–75]	[68–86]
		ACC (%)	54	71

**Fig. 6:**
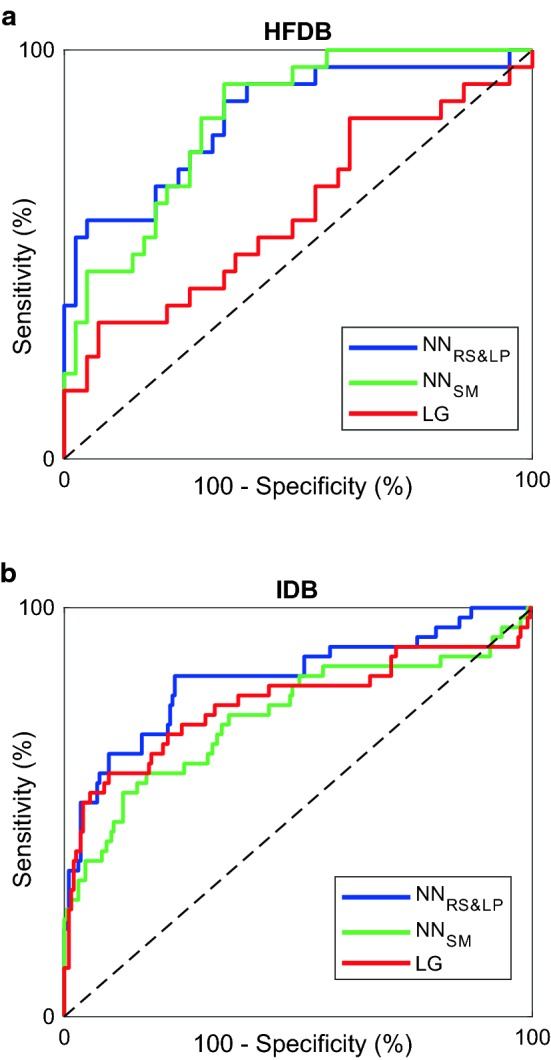
Receiver operating characteristics (ROCs) of the test results obtained with the neural networks with the RS&LP (NN_*RS&LP*_-blue lines), with the neural networks learnt with the standard method (NN_SM_-green lines) and with the logistic regression (LR-red lines) in the heart failure database (HFDB-**a**) and in the ischemia database (IDB-**b**)

Table 3 also shows the $\text {NN}_{SM}$ and LR results. $\text {NN}_{SM}$ performance ($AUC\ge {73\%}$; $ACC\ge {67\%}$ ) and LR performance ($AUC\ge {61\%}$; $ACC\ge {54\%}$) was inferior to $\text {NN}_{RS \& LP}$ performance for both databases. This finding is visualized in Fig.  6, where ROCs regarding $\text {NN}_{RS \& LP}$ were generally above ROCs regarding $\text {NN}_{SM}$ and LR. Superiority of NN over LR was statistically significant only in the IDB ($P<0.05$).

## Discussion

The present work presents a novel application of deep-learning NN classification to serial electrocardiography. Differently from current rule-based serial electrocardiography algorithms, our deep-learning approach considers several input features that likely vary (independently or in a relative fashion) during emerging or aggravating of any cardiac pathology.

Core of the here presented deep-learning NN approach is the new RS&LP, which dynamically creates a specific NN for a specific problem by iterative alternation of structuring and learning, while retaining the learning effect of the previous iteration in each new structure. This allows for reaching an efficient NN configuration without losing its generalization properties. RS&LP overcomes the problem that the standard learning procedures are only training NNs with fixed, user-defined architectures, since it consists of a systematic and controlled NN construction method that, additionally, integrates a weight-correction algorithm to adjust for disproportion between classes. The latter is likely occurring in clinical applications in which the number of controls is typically higher than the number of cases, which is also the case in our databases. Although originally designed for serial electrocardiography, RS&LP is a potentially useful tool in several other (not further specified to avoid speculation) classification problems, in medicine and other fields.

AUCs were chosen as performance index for all algorithms; indications of diagnostic ACC were computed at the points on the ROC where sensitivity equals specificity. Indeed, in clinical practice, the choice of an operating point on a ROC is a tradeoff between false-positive and false-negative decisions and associated costs. RS&LP yielded 3-layer NN architectures with high learning and testing performances (Table 3). Due to the limited sizes of testing datasets (65 and 241 ECG pairs for the HFDB and the IDB, respectively), CI remained relatively wide (22% and 16% for HFDB and IDB, respectively; Table 3). Neuron weight and bias values are available in Additional file 1 (NeuronWeightAndBias.mat).

For performance assessment of the RS&LP, we compared the results obtained with the $\text {NN}_{RS \& LP}$ against those obtained with the standard method to learn the NN ($\text {NN}_{SM}$) and against conventional LR, constructed on the same databases. In all cases, $\text {NN}_{RS \& LP}$ classification was superior to $\text {NN}_{SM}$ and to LR classification (Table 3, Fig. 6). The RS&LP provides better classification performances than standard NN learning; moreover, its property to construct the NN architecture during learning overcomes one of the challenges of NNs: the definition of the architecture. Future studies will evaluate the robustness of the chosen criteria, such as the maximal number of hidden layers or the number of iterations.

In an earlier study of our group on heart failure [16], ROCs were constructed by applying a variable threshold to signed and unsigned QSR-T spatial-angle differences; resulting AUCs were 72% and 78%, respectively. Another study on ischemia [20] compared performances of absolute differences of VG and ST-elevation, obtaining AUCs of 88% and 91%, respectively. Both studies [16, 20] were transversal analyses, performed on entire databases not split into learning and testing datasets; hence, no predictions can be made based on those results. AUCs of these studies have to be compared to our learning AUCs and not to our testing AUCs, which rather represent predictions. Our learning AUCs were all close to one (Table 3), thus higher than those in [16, 20]. Moreover, our testing AUC in the HFDB is 84%, which means that NN-based prediction outperforms the transversal classification in [16]. Similarly, our testing AUC in the IDB was 83%, very close to the transversal classification in [20].

Based on our results, we can conclude that our RS&LP yielded high-performing NNs readily applicable to serial ECGs to recognize emerging heart failure in post-infarction patients and acute ischemia in patients with a sudden short-lasting complete coronary occlusion. Still, other clinical applications in heart failure and ischemia require additional research. In emerging heart failure, serial ECG changes might already occur in the subclinical stage; if confirmed, serial ECG analysis could be used as a screening method in post-infarction patients. Ischemia detection by serial ECG analysis is of paramount importance in the real-world ambulance scenario, when patients are transported because of chest pain possibly related to acute coronary ischemia, possibly leading to a myocardial infarction. In this application, the FUECG is recorded in the ambulance, whereas the BLECG is to be found in ECG databases of hospitals and may be several years old. Compared to our case patients, case ambulance patients mostly suffer from acute coronary syndrome, which can manifest in various forms. For example, occlusions may be dynamic and may have been present much longer than the duration of the balloon inflations in the STAFF III database. The classification problem is further complicated because the control ambulance patients (those with no ischemia) may have other acute ECG-affecting pathologies, like pulmonary embolism or pericarditis. Thus, ECG changes measured in ambulance patients will be different from those observed in our IDB patients, and a specific NN needs to be constructed on the basis of serial ECGs that represent the specific mix of patients with ischemia (cases) and patients without ischemia, but often with other pathology (controls), as they present themselves to the emergence medical services.

## Conclusion

In conclusion, although we cannot claim that our method is universally suited for the construction of problem-specific NNs for serial ECG comparison, we consider it as a strength that it was successful in two very different clinical applications: the detection of newly emerging heart failure in post-infarction patients, and the detection of acute ischemia. Further exploration of our method has to reveal if other problem-specific NNs can successfully be constructed, and even if it will be possible to construct a universal NN to detect any pathologic change in the ECG.

## Additional file

**Additional file 1.** NeuronWeightAndBias.mat is a Matlab file, that contains the Weights and Biases of the neural network obtained bythe repeated structuring and learning procedure.

## Abbreviations

$|\Delta Jampl|$: magnitude of J vectors difference
$|\Delta VG|$: magnitude of ventricular-gradient difference vector
ACC: accuracy
AUC: area under the curve
BLECG: baseline electrocardiogram
CI: 95% confidence interval
ECG: electrocardiogram
FUECG: follow-up electrocardiogram
HFDB: heart-failure database
IDB: ischemia database
LR: logistic regression
NN: neural network
$\text {NN}_{RS \& LP}$: neural network obtained with the repeated structuring and learning procedure
$\text {NN}_{SM}$: neural network obtained with the standard method
ROC: receiver-operating characteristic
RS&LP: repeated structuring and learning procedure
VCG: vectorcardiogram
$\Delta HR$: heart-rate difference
$\Delta QRScmplx$: QRS-complexity difference
$\Delta QRSdur$: QRS-duration difference
$\Delta QRSintg$: QRS-integral vector magnitude difference
$\Delta QRSmax$: maximal QRS-vector magnitude difference
$\Delta QTint$: QT-interval difference
$\Delta Tcmplx$: T-wave complexity difference
$\Delta Tintg$: T-integral vector magnitude difference
$\Delta Tmax$: maximal T-vector magnitude difference
$\Delta Tsym$: T-wave symmetry difference
$|\Delta SA|$: spatial-angle absolute difference
